# Polysaccharides derived from *Balanophora polyandra* significantly suppressed the proliferation of ovarian cancer cells through P53‐mediated pathway

**DOI:** 10.1111/jcmm.15468

**Published:** 2020-06-10

**Authors:** Jiayuan Qu, Yumin He, Yue Shi, Liyue Gai, Li Xiao, Fan Peng, Zicheng Li, Xiaomin Wang, Chengfu Yuan

**Affiliations:** ^1^ College of Medical Science China Three Gorges University Yichang China; ^2^ Third‐Grade Pharmacological Laboratory on Chinese Medicine Approved by State Administration of Traditional Chinese Medicine Medical College of China Three Gorges Yichang China; ^3^ Chumeiren Medical Cosmetic Clinic Yichang China; ^4^ Hubei Key Laboratory of Tumour Microenvironment and Immunotherapy China Three Gorges University Yichang China

**Keywords:** apoptosis, *Balanophora polyandra*, cell cycle arrest, migration and invasion, ovarian cancer, P53

## Abstract

Ovarian cancer (OC) is ranked the first among the cancers threatening women's health. It attracts tremendous attention of cancer researchers because of its extremely high mortality rate. Recent studies have indicated that traditional herbal medicines (THMs) can play a pivotal role in cancer prevention and treatment. THMs are gaining popularity as a source of anti‐cancer agents. The plant of *Balanophora polyandra*, which has been used as a traditional herbal medicine, has been known for exhibiting potential haemostatic, analgesic, anti‐inflammatory and anti‐cancer properties. However, few studies on inhibitory effect of *B. polyandra* on OC have been performed. In the present study, we found that *B. polyandra* polysaccharides (BPP) induced cell cycle arrest at S phase, triggered apoptosis and inhibited migration and invasion of OC cells. Furthermore, we also found that there was a potential and close relationship between BPP and P53‐mediated pathway. Overall, these findings suggest that BPP can be a potential therapeutic agent for the treatment of OC.

## 1 INTRODUCTION

Uncontrolled proliferation is known to be one of the most important cancer cell hallmarks and plays critical roles in carcinogenesis and progression in malignant tumours. Among gynaecological cancer, ovarian cancer (OC) is a leading driver of cancer‐related mortality in women, according to the systematization global examination of the disease.^1^ While the OC incidence has remained relatively static over the last 35 years,^2^ its survival rates are still low, partly because of its resistance to the currently existing therapies. About 75% of patients with OC responded well to the standard first‐line chemotherapy treatment, but soon OC usually returns and develops resistance to the previous chemotherapy agents. Therefore, discovery and development of new anti‐OC therapeutic agents is urgently and highly demanded for improving the clinical outcome.

Traditional herbal medicines (THMs) have been applied as the important components of complementary and alternative medicine. They can be used to treat more than 100 diseases.^3^ In the recent years, THMs are gaining popularity because of their significant role in preventing and treating various types of cancer, with advantages of preventing tumorigenesis, enhancing the therapeutic effect as well as attenuating toxicity.^4^ In previous reports, several kinds of THMs have been shown to possess prospective anti‐cancer properties, and some bioactive components from THMs have been used for clinical therapy for several types of diseases, such as Kanglaite, artemisinin, podophyllotoxin and paclitaxel.^5^, ^6^, ^7^, ^8^ Those research literatures indicate that HTMs can have a great potential to be developed into a new type of anti‐ovarian cancer agents.

*Balanophora polyandra* (*B. polyandra*), belonging to the family Balanophoraceae, is a native parasitic plant that grows on the root system of various hosts, especially the evergreen vine shrubs in south‐west of China.^9^ It has been traditionally used as a medicinal plant for treatment of various diseases, including haemorrhoids, metrorrhagia, stomach ache and hemoptysis. Phytochemical studies on the whole plant of *B. polyandra* have reported the presence of hydrolysable tannins, particularly the ellagitannins, which are considered to be the characteristic components in genus Balanophora.^10^ In addition, other components like phenylpropanoids, flavonoids, terpenoids and polysaccharides are also existed.^11^ Previous studies have revealed that the polysaccharides derived from *B. polyandra* possessed anti‐fatigue, antioxidant and hypoglycaemic activities, whereas the polysaccharides from B. spicata could efficiently inhibit the growth of the implanted S180 tumour.^12^ However, there have been no studies on its roles in other types of cancers, such as OC, and the underlying mechanisms.

Herein, we assessed how *B. polyandra* polysaccharides (BPP) inhibit the growth of OC cells. We found that BPP was significantly related with proliferation, apoptosis, cell cycle arrest, invasion and migration of OC cells. Moreover, we gained some insight into the mechanisms by which BPP inhibits the proliferation of OC cells. We also found that P53 pathway was involved in the induction of the cell cycle arrest and apoptosis in vitro. These findings suggest that BPP can have the potential to inhibit OC progression.

## 2 MATERIALS AND METHODS

### 2.1 Cells culture

Two human ovarian cancer cell lines, A2780 and OVSAHO, came from Cell Resource Center of Shanghai Institutes for Biological Sciences (Shanghai, China). These cells were grown with DMEM containing 10% FBS and 1% penicillin/streptomycin. All the cells were grown in a 5% CO_2_ incubator at 37°C. Cells were used when they were in the logarithmic growth phase.

MTT, crystal violet and propidium iodide (PI) were obtained from Sigma‐Aldrich (St. Louis, MO, USA). Annexin V‐FITC/PI was purchased from Bestbio (Bestbio, Shanghai, China), and all primary antibodies were purchased from Wanleibio (Wanleibio, Shenyang, China). Matrigel™ Basement Membrane Matrix was from BD Biosciences (San Jose, CA, USA). All the primer sequences were synthesized at Sangon Biotech (Shanghai) Co., Ltd.

### 2.2 Plant material

Whole *B. polyandra* plants were obtained from Wufeng county of Hubei province (China) in 2016. A voucher sample (2W16080501) has been deposited in Third‐Grade Pharmacological Laboratory on TCM with approval from the State Administration of Traditional Chinese Medicine (SATCM). All the other chemical and reagents used for the preparation and analysis of BPP were of analytical grade from China.

### 2.3 Preparation of polysaccharides and measurement of carbohydrate content

The dried whole plant of *B. polyandra* (300 g) was firstly minced into small pieces, then extracted using 2.0 L of distilled water at 95°C for 2.5 hours thrice and finally filtered through a multi‐layer gauze. After centrifugation at 1500 *g* for 10 minutes, the supernatant was saved and concentrated to 1.5 L, and precipitated with 95% ethanol (2.5 volumes) overnight at 4°C. The pellet was separated by centrifugation, then dissolved in distilled water and treated with Sevag reagent (4:1 of chloroform/‐butanol) to remove proteins. The deproteinized solution was lyophilized, and light brown polysaccharide extract (26.5 g) was obtained. The total carbohydrate content of the polysaccharide extract was determined with phenol‐sulphuric acid methods to be 54.0% (g/g) and expressed as anhydrous glucose equivalent.

### 2.4 MTT assay

Both A2780 and OVSAHO cells were added to 96‐well plates (2000 per well). The next day, compounds were diluted to different concentrations with media supplemented using 5% FBS before addition to appropriate wells. After incubated for 24, 48 and 72 hours, the cell viability was detected via incubation in a 1 mg/mL MTT solution in media for 4 hours, respectively. Next, formazan was solubilized through addition of DMSO (100 μL) for 10 minutes, shaking while protected from light. Finally, absorbance 570 nm (OD_570_) was assessed via microplate reader (Thermo Electron, TYPE1500‐458, Waltham, MA, USA).

### 2.5 Crystal violet staining assay

A2780 and OVSAHO cells were plated at 2 × 10^4^ cells/well in a 12‐well culture plates and cultured in 37°C incubators with a DMEM medium containing 5% newborn calf serum and 1% antibiotics. A medium containing BPP at different concentrations was added for 72 hours. The crystal violet solution was added to cover the cell surface and incubated for 20‐30 minutes. The residual crystal violet was washed away with deionized water, noting that water was not dropped directly to cells. The plate was air‐dried and photographed with scanner. Then, the crystal violet was solubilized with 1% SDS reagent and the concentration of the colorimetric probe was determined by an optical density measurement at 570 nm.

### 2.6 RNA extraction and cDNA analysis by qPCR

Quantitative PCR (qPCR) was applied to analyse the mRNA expression levels of cyclin A, CDK2, Bax, Bal‐2, P53 and P21 in OC cells samples. The sequences of the primers are listed in support data. Total RNA samples were extracted from A2780 and OVSAHO cells using TRIzol (Takara, Cat. 9109), following the protocol. An aliquot of RNA from A2780 and OVSAHO cells was reverse‐transcribed via M‐MLV Reverse Transcriptase (New England Biolabs, Cat. M0251S) and random hexamers, following the protocol. The primers were synthesized by Sangon Biotech (Shanghai, China). Amplification and melt curve analysis were performed using an qTOWER 3.0 system (Analytik Jena, Jena, Germany). SYBR^®^ Premix Ex Taq kit (Takara, Dalian China) was used for reactions, and total reaction volume was 10 μL. The 2^−ΔΔCt^ method was used for quantifying relative expression.

### 2.7 Western blotting

Protein samples were prepared from untreated or treated OC cells and first separated by SDS‐polyacrylamide gel electrophoresis (SDS‐PAGE) and then transferred to PVDF membranes. The proteins of interest were probed by incubation with the primary antibody (1000 times dilution) at 4°C overnight. The membrane was washed three times (10 min/time) in TBST with gentle agitating to remove residual primary antibody and incubated with corresponding secondary antibody for 1 hour at 37°C. The membranes were washed and imaged by using an ECL chemiluminescent substrate kit according to the instructions provided (Thermo Scientific).

### 2.8 Cell cycle analysis by flow cytometry

OC cells treated using serially diluted BPP for 48 hours were fixed in ice‐cold 70% ethanol for 4 hours at 4°C. After washed two times in PBS, cells were resuspended in citrate buffer with RNase A and propidium iodide (10 ng/mL and 50 μg/mL) and incubated for 30 minutes at 37°C. The cell cycle was analysed with the flow cytometry (FACS CantoTM, BD Bioscience, Franklin Lakes, NJ, USA). Data were analysed using ModFit 161 LT version 3.0 software (Verity Software House).

### 2.9 Apoptosis assay by flow cytometer

Apoptosis in cells was induced by using serial concentrations of BPP for 48 hours with no BPP sample used as a negative control. After incubation, the cells were harvested and washed in cold PBS. Next, annexin V and PI (Bestbio, Shanghai, China) were used for staining at room temperature for 15 minutes and the stained cells were analysed immediately by flow cytometry (FACS CantoTM, BD Bioscience, Franklin Lakes, NJ, USA) to assess apoptotic cell frequency.

### 2.10 Wound healing assay

OC cells were placed in 6‐well plates and cultured until full confluence. A 10 μL pipette tip was used to make a straight scratch, simulating a wound, and the wells were gently washed two times using PBS to remove the detached cells. After scratching, serum‐free medium containing BPP at 0, 20, 40 and 60 μg/mL serial concentrations was added, respectively. Images of the wound area were obtained at 0, 24 and 48 hours. ImageJ was used to measure the scratch area in the visual field for statistical analysis.

### 2.11 Migration assay

The Matrigel with serum‐free DMEM medium formed 1:7 complex and then vertically added to a chamber (50 μL/well) at 37°C for 4 hours to be coagulated. A mix of 0.6 mL of DMEM containing 10% newborn calf serum was added in the lower compartment, after which a Transwell insert was put in place. Cells were grown in serum‐free medium for 24 hours, after which 2 × 10^4^ cells treated with BPP at different concentrations were gently put in the upper compartment and incubated for 48 hours. Thereafter, the chamber out was taken out and the cells in the chamber were removed via cotton swab, and then, crystal violet solution was added to cover the cell surface for 20‐30 minutes. The cells were observed, and photographs were taken under optical microscope. Then, the crystal violet was solubilized with the 1% SDS reagent, and the concentration of the colorimetric probe was determined by an optical density measurement at 570 nm (OD_570_). Each experiment was repeated three times.

### 2.12 Animals and treatment

All the animal experiments were consistent with the ‘Guide for the Care and Use of Laboratory Animals’, with the China Three Gorges University Animal Experimental Ethics Committee approving these studies. Female BALB/c nu/nu mice (approximately 16 g, 5 weeks old) were purchased from Beijing Vital River Laboratory Animal Technology Co., Ltd. Animals were given 1 week to acclimate. The cell suspensions in log phase of human ovarian cancer of A2780 cell line were implanted into the dorsal skin of nude mice to develop a tumour model. Then, these nude mice were randomly separated into control group (only given NS), low‐dose group (200 mg/kg·d BPP) and high‐dose group (400 mg/kg·d BPP). These nude mice were intragastrically administrated once a day. The parameters of the longest and the shortest diameter were observed by vernier caliper on 7, 14, 21 and 28 days after treatment. The tumour volume was calculated according to the calculation formula of V = length × width^2^ × *π*/6. After being treated for 28 days, the tumour weight was measured and the inhibitory rate of tumour growth was calculated.

### 2.13 Statistical analysis

The experimental results were means ± SD. The statistical software SPSS 20.0 was used for analysis, and one‐way ANOVA was used for multi‐group analysis. All the results were repeated at least three times.

## 3 RESULT

### 3.1 BPP inhibits ovarian cancer cell proliferation

**FIGURE 1:**
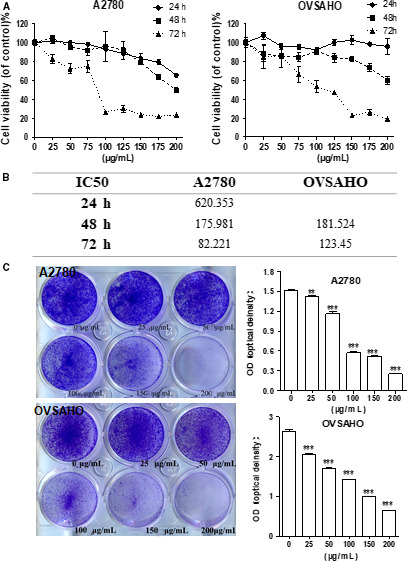
BPP suppresses proliferation of ovarian cancer cells in vitro. MTT experiments (A) and crystal violet (C) to show the inhibitory effect on the growth/proliferation of BPP on OVSAHO and A2780 cells after treatment with BPP at different concentrations. Representative images are shown. B, 50% inhibitive concentration (IC_50_) of BPP was examined. Data are means ± SD from three experiments. The experiments were repeated at least three times. *P < 0.05 compared with the control; **P < 0.01 compared with the control; ***P < 0.001 compared with the control

To study whether BPP inhibits OC cell proliferation, we used MTT to test its proliferation‐inhibiting effect of human OC cells, A2780 and OVSAHO treated with BPP. We found that at each time‐point, the proliferation rates of the OC cells were significantly increased (*P < *0.005) with increasing BPP concentration. In addition, the cell viability of the OC cells was significantly decreased as time was prolonged (*P < *0.005) (Figure 1A). As shown in Figure 1B, the IC_50_ values of BPP on A2780 and OVSAHO were gradually decreased with prolonging the treatment time and increasing concentration of BPP. These experimental results showed that BPP inhibited the proliferation of OC cells in a dose‐ and time‐dependent manner.

Then, we choose the concentrations of BPP at 50, 100 and 150 μg/mL for treatment of A2780 and OVSAHO cells for 48 hours. Surviving cells were fixed and stained with crystal violet, and the OD570 was measured. As shown in Figure 1C, with the concentration of BPP increased, the number of the cells significantly reduced with increasing BPP concentration. These results suggest that BPP is capable of inhibiting the proliferation of the two lines of OC cells.

### 3.2 BPP inhibited the invasion and migration on ovarian cancer cells

**FIGURE 2:**
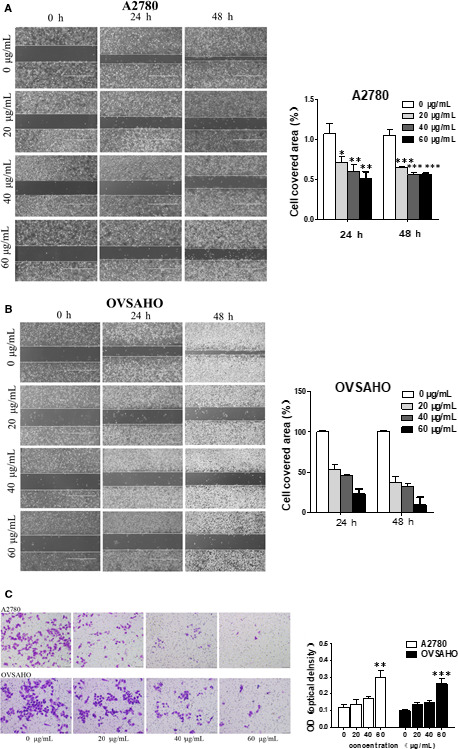
BPP inhibits A2780 and OVSAHO mobility. A2780(A) and OVSAHO(B) cell migration was assessed via wound healing assay after treatment with BPP at different concentrations. The area covered with cells was used for quantifying migration rats. C, Transwell method was used to detect the invasiveness of the cells and the absorbance of the stained cells was detected after elution. Data are means ± SD of triplicate experiments. *P < 0.05 compared with the control; **P < 0.01 compared with the control; ***P < 0.001 compared with the control

To discover whether BPP is capable of suppressing the invasion and migration of A2780 and OVSAHO OC cells in vitro, we conducted the wound healing assay and Transwell invasion assay to assess its effects on the invasion and migration of A2780 and OVSAHO cells after treatment with BPP at 20, 40 and 60 μg/mL. In wound healing assay (Figure 2A), migration (the open space) of A2780 cells was gradually reduced to 71.39% (20 μg/mL), 59.77% (40 μg/mL) and 51.05% (60 μg/mL) after 24 hours treatment with BPP and to 64.79% (20 μg/mL), 55.53% (40 μg/mL) and 56.25% (60 μg/mL) after 48 hours treatment with BPP, respectively. For OVSAHO cells (Figure 1B), the cell migration rate was decreased to 52.81% (20 μg/mL), 45.78% (40 μg/mL) and 23.32% (60 μg/mL) after 24 hours treatment with BPP and to 37.61% (20 μg/mL), 32.35% (40 μg/mL) and 9.54% (60 μg/mL) after 48 hours treatment with BPP, respectively. These data indicate that the migration capability of OC cells is significantly reduced by BPP.

Having demonstrated the capability of BPP in inhibiting invasion and migration, we further investigated its effects on cell invasiveness with a Transwell assay. As shown in Figure 2C,D, BPP was capable of significantly inhibiting the invasion ability and collagenolytic activity of A2780 and OVSAHO in an obvious dose‐dependent manner. Taken together, our data indicate that the migration and invasion abilities could be suppressed by BPP at low concentration, and the abilities of migration and invasion were reduced constantly with increasing BPP concentration.

### 3.3 BPP induced apoptosis on ovarian cancer cells

**FIGURE 3:**
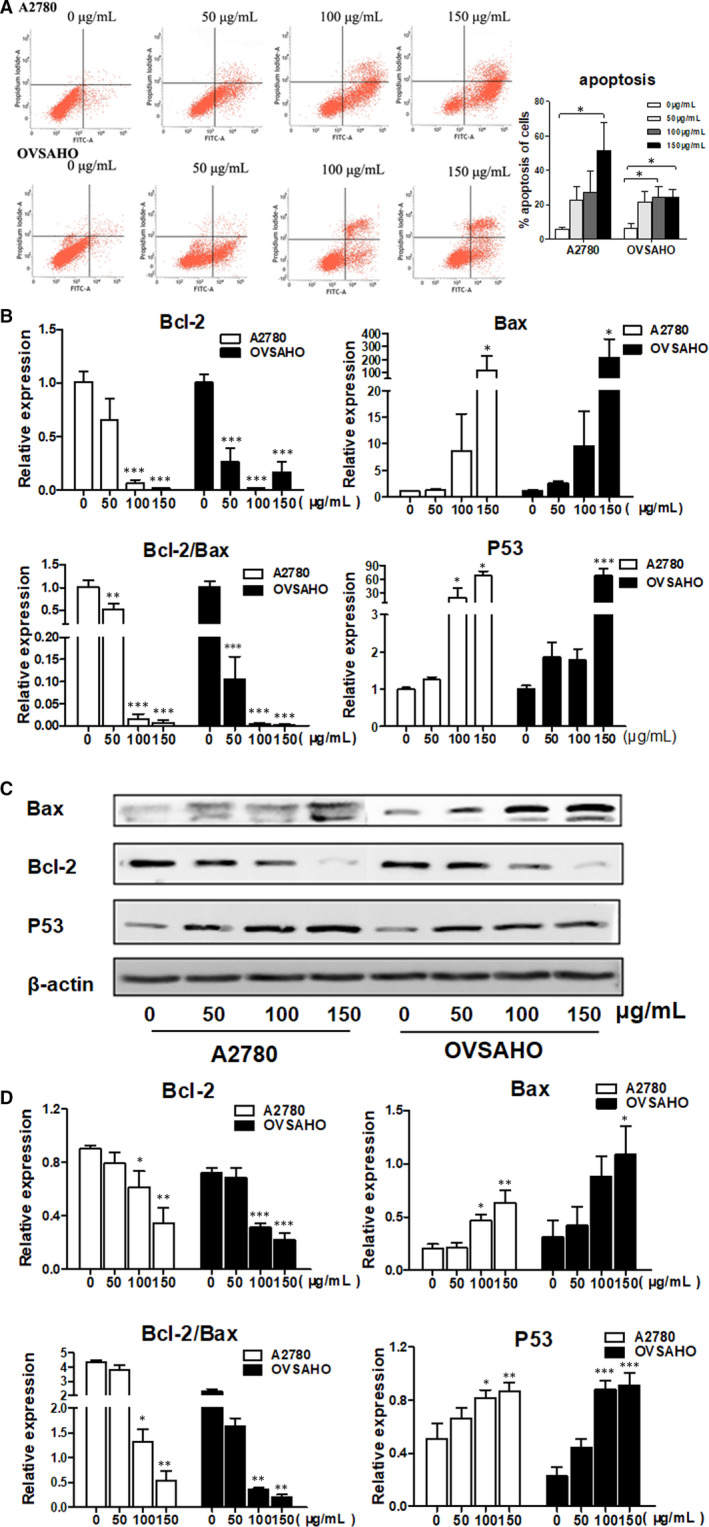
BPP promotes apoptosis of ovarian cancer cells in vitro. A, Annexin V/PI staining was used for measuring apoptosis using a flow cytometer. A2780 and OVSAHO cells were treated with BPP at different concentrations for 72 h, and cell cycle distribution was analysed by flow cytometry; Bax, Bcl‐2, P53 and Bcl‐2/Bax expression was determined by qPCR (B) and Western blot (C & D). Data are presented as mean ± SD. The experiments were repeated at least three times. *P < 0.05 compared with the control; **P < 0.01 compared with the control; ***P < 0.001 compared with the control

It has been known that apoptosis is an important component mediating suppression of cell proliferation. In the present study, we estimated apoptosis after BPP treatment based on Annexin V/PI staining and assessment via flow cytometry. Compared with that of the negative control, the total apoptotic cells were significantly increased from 5.963% to 51.50% in A2780 cells and 6.557% to 24.48% in OVSAHO cells after BPP treatment at 150 μg/mL for 48 hours (*P < *0.05), respectively (Figure 3A). Moreover, the expression levels of apoptosis‐related gene Bax were significantly increased in A2780 and OVSAHO cells after being treated with BPP. Both qPCR analysis and Western blot revealed that the expression levels of Bcl‐2 were dramatically lowered after BPP treatment (Figure 3B‐D). With increasing the drug concentration, the ratio of Bcl‐2/Bax was dropped progressively and apoptosis cells appeared. Our data thus show that BPP is capable of inducing apoptosis in A2780 and OVSAHO cells.

### 3.4 BPP arrested cell cycle progression in S phase

**FIGURE 4:**
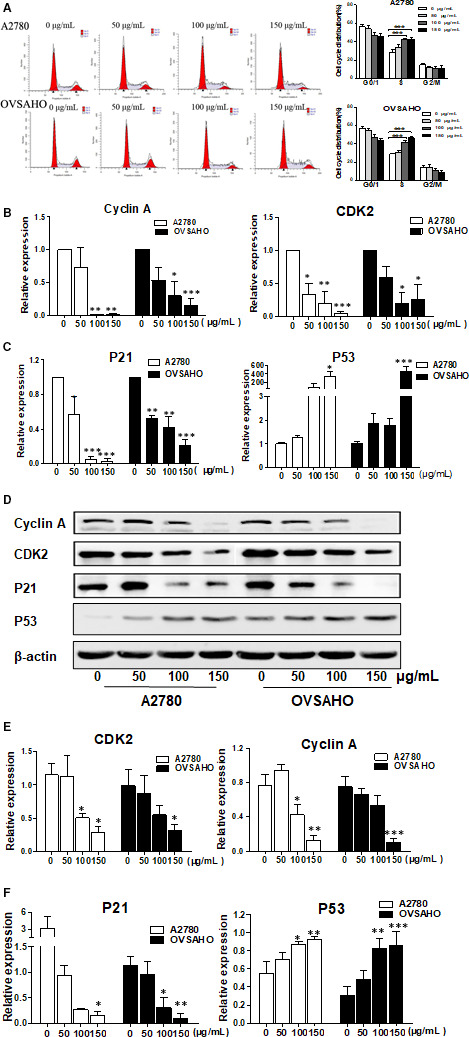
BPP changes OC cell cycle distribution in vitro. A, A2780 and OVSAHO cells were treated using BPP at different concentrations for 72 h, and the cell cycle distributions were measured; the expression levels of cyclin A, CDK2, P21 and P53 were examined by qPCR (B&C) and Western blot (D&E). Data are presented as mean ± SD. The experiments were repeated at least three times. *P < 0.05 compared with the control; **P < 0.01 compared with the control; ***P < 0.001 compared with the control

To explore the possible mechanism underlying the inhibition of cell proliferation activity of BPP towards A2780 and OVSAHO cells, we estimated the changes in cell cycle phases using flow cytometry analysis. As shown in Figure 4A, treatment of A2780 and OVSAHO cells with BPP at concentrations of 50, 100 and 150 μg/mL for 48 hours induced significantly S‐phase arrest in a concentration‐dependent manner. The S‐phase arrest rates of A2780 and OVSAHO cells treated with the highest BPP concentration (150 μg/mL) tested were significantly increased from 28.40% to 42.95% and from 26.49% to 41.23%, respectively, as compared with those of the negative control group (*P* < 0.001).

As we know, cyclin A‐kinase function is essential for S‐phase progression; therefore, we examined the expression levels of cyclin A and the corresponding cyclin‐dependent kinase 2 (CDK2).^13^ As shown in Figure 4B,D,E, the expression levels of both cyclin A and CDK2 were significantly down‐regulated in BPP‐treated A2780 and OVSAHO cells at both mRNA and protein levels. These data indicate that BPP can induce S‐phase cell cycle arrest in both A2780 and OVSAHO cells.

### 3.5 BPP affected the P53 signalling pathway

As described above, BPP is capable of inducing S cell cycle arrest, triggering apoptosis and preventing OC cells invasion and migration. However, the underlying mechanism is poorly understood. It is well known that P53 is one of the most important genes associated with human tumours, which can influence tumour proliferation by mediating both apoptosis and cell cycle. Therefore, we assessed the mRNA levels and protein expression levels of both P53 and P21 in OC cells and nude mouse models. As shown in Figure 4C,F, P53 expression was increased, whereas P21 was down‐regulated by BPP in a dose‐dependent fashion. Altogether, these data revealed that BPP has strong anti‐tumour effects, which are related with P53 pathway‐mediated apoptosis and S cell cycle arrest.

### 3.6 BPP significantly suppresses ovarian tumour growth in vivo

**FIGURE 5:**
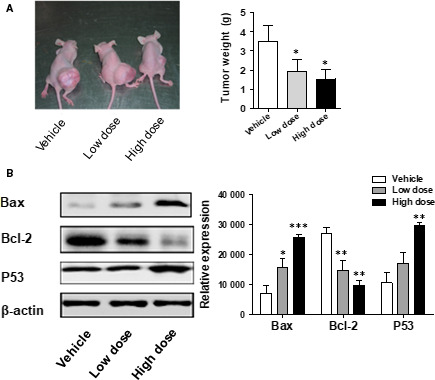
BPP treatment reduces the growth and metastasis of tumours in vivo. A, Images of excised tumours 28 d after treatment by BPP. B, Bax, Bcl‐2 and P53 levels measured by Western blotting. *P < 0.05 compared with the control; **P < 0.01 compared with the control; ***P < 0.001 compared with the control

Having shown that in vitro BPP can inhibit the proliferation and induce the apoptosis of OC cells, we then conducted the experimentation to examine the anti‐tumour effect with BPP recombinant antibodies in vivo. As shown in Figure 5A, BPP suppressed the growth of transplanted tumour from ovarian neoplasm nude mice after 28 days of intragastric administration, and the tumour weight was significantly reduced (*P* < 0.05). And in low‐dose and high‐dose groups, the suppression rates of xenograft tumour growth were 36.06% and 32.56%, respectively. And then, Bax, Bcl‐2 and P53 protein levels in the normal group, the low‐dose group and the high‐dose group were also examined by Western blot. Relative to the control group, the expression levels of Bax (Figure 5B) and P53 were significantly increased and Bcl‐2 was decreased when dose of BPP was increased (*P < *0.05). Together, this indicates that BPP is capable of inhibiting the growth of ovarian tumour by inducing apoptosis via P53 signalling pathways in vivo.

## 4 DISCUSSION

OC is ranked the first among the cancers threatening women's health. It has attracted tremendous attention of cancer researchers because of its extremely high mortality rate. At the present, radiotherapy and chemotherapy are widely used in clinic. However, their side effects are relatively serious. The use of THMs as adjuvant therapy to alleviate cancer symptoms has advantages over Western medicine in specific aspects, such as prevention of tumorigenesis, attenuation of toxicity and enhancement of the therapeutic effect. Thus, OC has become a hot research and development object of anti‐cancer drugs.

BPP from *Balanophora* plants has the effects of haemostatic, analgesic, anti‐inflammation and anti‐tumour.^14^, ^15^, ^16^ BPP is extracted from the whole plant of *B. polyandra* by the methods involving hot‐water lixiviation, ethanol sedimentation and Sevag's deproteinization. However, at the present, there have been no reports on the treatment of ovarian cancer with BPP. Herein, we reported for the first time that BPP has an inhibitory effect on the growth of OC cells and xenograft tumours in a dose‐dependent manner. In addition, BPP can inhibit cell invasion and migration, induce cancer cell apoptosis and block cell cycle, which might be resulted from the BPP multiple targets of complex diseases, including ovarian cancer.

To investigate the influence of BPP on the invasion and migration of OC cells, the wound healing assay and migration assay were used to observe the changes of the migration and invasion of OC cells under the action of BPP, respectively. Because the results of MTT experiment showed that when the BPP concentration was above 60 μg/Ml, the cell survival rate of OC cells is <80% at 48 hours. To avoid the proliferation inhibition of BPP on OC cells leading to false‐positive results of the wound healing assay and migration assay, 20, 40 and 60 μg/mL of BPP were selected for subsequent studies. The results, shown in Figure 2A,B, indicate that BPP has significant effects of anti‐migration and anti‐invasion on A2780 and OVSAHO cells.

Apoptosis is a programmed death which is highly regulated. It has been shown by relevant studies that the occurrence, development and regression of OC are closely related to apoptosis, so inducing apoptosis of cancer cells has become a hot spot and a new target for tumour treatment.^17^ In this study, we used Annexin V/PI staining to assess OC cell apoptosis induced by BPP and found that BPP could significantly induce apoptosis of human ovarian cancer cells A2780 and OVSAHO. Both Bcl‐2 and Bax play important regulatory roles in apoptotic pathways.^18^ When Bax is highly expressed, cells are sensitive to death signals and promote apoptosis; when Bcl‐2 is highly expressed, Bcl‐2 and Bax can form heterodimers and inhibit apoptosis.^19^ Therefore, the relative expression levels of Bcl‐2 and Bax in cells and the ratio of Bcl‐2/Bax are the very important indicators for determining the sensitivity of cell apoptosis. In this study, we found that BPP could significantly inhibit Bcl‐2 expression in both OC cells and xenograft tumours in nude mice, and significantly rose the expression level of Bax. The decrease in Bcl‐2/Bax ratio suggested that BPP could significantly activate apoptosis in ovarian cancer.

The mitotic intervals in cell cycle can be divided into three stages: DNA pre‐synthetic stage (G1 stage), DNA synthesis stage (S stage) and DNA post‐synthetic stage (G2 stage). PI staining assay indicated that the DNA synthesis of ovarian cancer cells was inhibited after BPP treatment and that the cell cycle was blocked at S stage in a dose‐dependent manner. Research by Nurse et al^20^ found that cyclin‐dependent kinase (CDK) plays an important role in the cell cycle, which is regulated the cell cycle by chemically modifying other proteins; Timothy Hunt demonstrated that cyclin is cyclically degraded during each cell division and regulates the cell cycle by regulating CDK.^21^ Recent researches have found 8 types of cyclin, cyclin A‐H, in mammalian cells.^22^ In the process of cell cycle regulation, cyclin proteins that are cyclically expressed bind to CDK proteins to form complexes, thereby activating the kinase activity of CDK and regulating the cell cycle. Relevant studies have shown that if the expression of cyclin A is inhibited in cells, resulting in the down‐regulated expression of the bound CDK2, the cell cycle will be stagnant in the S phase.^23^, ^24^ As shown in Figure 4, BPP can inhibit the formation of cyclin A‐CDK2 complex by inhibiting their expression and, thus, mediates cell cycle arrest in S stage.

P53 signalling is important in the development of cancer. It controls a wide and flexible signalling networks and regulates the genome integrity. There are two ways for P53 to participate in the repair of cell damage as follows: first, P21 gene promoter region has p53 binding site, so p53 can bind to it and activates p21 gene transcription.^25^ When the DNA of cells is damaged, the expression level of p21 gene is increased under the induction of p53, and the CDK activity is inhibited; thus, the cell cycle is affected. Second, when DNA damage is too serious to be repaired, P53 can induce Bax expression, thus regulating cell apoptosis.^26^ In this experiment, we explored whether BPP affected P53 signalling pathway and found that the changes in P53 and P21 activities were correlated with the proliferation of OC cells and tissues stimulated by BPP, suggesting that P53 may be one of the important signalling pathways in the process of BPP regulating the proliferation of OC cells.

At the present, the continuous exploration of the inhibition of BPP on the proliferation of cervical cancer has provided new ideas and directions for pro‐drug screening and treatment of ovarian cancer. However, there are still some defects in the current research on the treatment of BPP on ovarian cancer, and the main aspects are as follows: (a) while BPP showed a moderate to strong inhibitory activity against ovarian cancer cells, its specific chemical and structural properties are still unclear and need to be further studied; (b) the present anti‐cervical cancer activity experiment of BPP is still at a relatively shallow level, and there are few studies on the related mechanism, which is urgent to be further elucidated at present and in the future stage; and (c) the effect of BPP combined with surgery or radiotherapy and chemotherapy has not been studied yet. It is hopeful to explore a new direction for the development and application of anti‐cancer drugs for clinical ovarian cancer by taking the advantages of BPP, such as multi‐target, lower toxicity and fewer side effects. Therefore, it is necessary to further study the anti‐cancer activity and the underlying mechanism of anti‐ovarian cancer drugs in order to achieve a deeper understanding of the mechanism and biomedicine of ovarian cancer.

## 5 CONCLUSION

This research has clearly revealed that BPP can inhibit proliferation ovarian cancer cells via inducing apoptosis, blocking cell cycle in S phase and inhibiting migration and invasion. The results also suggest that P53 pathway may be an important signal pathway involved in inhibition of proliferation of ovarian cancer. BPP can effectively prohibit the proliferation of ovarian cancer and, thus, can be a new medicine to be used for treatment of ovarian cancer.

## CONFLICT OF INTEREST

The authors declare that they have no conflict of interest.

## AUTHOR CONTRIBUTIONS

Jiayuan Qu contributions: writing (original draft) and data curation. Yumin He contributions: methodology and investigation. Liyue Gai, Yue Shi and Fan Peng contributions: formal analysis. Li Xiao, Zicheng Li and Xiaomin Wang contributions: supervision and project administration. Chengfu Yuan contributions: writing (review and editing) and funding acquisition. All authors read and approved the final manuscript.

## ETHICAL APPROVAL

All the animal experiments were consistent with the ‘Guide for the Care and Use of Laboratory Animals’, with the China Three Gorges University Animal Experimental Ethics Committee approving these studies.

## Data Availability

The data used to support the findings of this study are available from the corresponding author upon request.
